# Therapeutic Management in Patients with Chronic Obstructive Pulmonary Disease Who Are Overweight or Obese: A Systematic Review and Meta-Analysis

**DOI:** 10.3390/jcm14041230

**Published:** 2025-02-13

**Authors:** Sara Chami-Peña, Alberto Caballero-Vázquez, María José Mebrive-Jiménez, José L. Gómez-Urquiza, José L. Romero-Bejar, Antonio M. Caballero-Mateos, Guillermo A. Cañadas-De la Fuente

**Affiliations:** 1 Centro de Salud Ronda Norte, AGS Serranía de Málaga, Andalusian Health Service, 29400 Ronda, Spain; sarachamip@gmail.com; 2 Diagnostic Lung Cancer Unit, Broncopleural Techniques and Interventional Pulmonology Department, Hospital Universitario Virgen de las Nieves, 18014 Granada, Spain; 3 Faculty of Health Sciences, University of Granada, Cortadura del Valle s/n, 51001 Ceuta, Spain; 4 Department of Statistics and Operations Research, University of Granada, Av. de Fuente Nueva, s/n, 18071 Granada, Spain; jlrbejar@ugr.es; 5 Instituto de Investigación Biosanitaria (ibs. GRANADA), 18012 Granada, Spain; 6 Department of Gastroenterology, San Cecilio University Hospital, Andalusian Health Service, Av. del Conocimiento s/n, 18016 Granada, Spain; 7 Department of Internal Medicine, Gastroenterology Section, Santa Ana Hospital, Andalusian Health Service, Av. Enrique Martín Cuevas, s/n, 18600 Motril, Spain; 8 Faculty of Health Sciences, University of Granada, Av. Ilustración 60, 18016 Granada, Spain; gacf@ugr.es; 9 Brain, Mind and Behaviour Research Centre (CIMCYC), University of Granada, Campus Universitario de Cartuja s/n, 18011 Granada, Spain

**Keywords:** chronic obstructive pulmonary disease, meta-analysis, overweight, obesity, obesity paradox

## Abstract

**Introduction/Objective**: The relationship between chronic obstructive pulmonary disease (COPD) and overweight is complex and multifaceted, as these conditions can interact in terms of symptoms, severity and clinical management. To analyse the clinical and therapeutic management of patients suffering from COPD and overweight. **Methods**: This systematic review was carried out, in accordance with the PRISMA statement, during November 2024, following a search of the Medline/PubMed databases. The search equation used, with MESH descriptors, was: “(Pulmonary Disease, Chronic Obstructive OR COPD) AND (obesity OR overweight)”. Both inclusion and exclusion criteria were applied, focusing on the selection of clinical trials. The studies were classified into two main groups: by their focus on the relationship between overweight/obesity and COPD; and by the benefits provided by physical exercise to patients with these conditions. A random-effects meta-analysis was performed on the data obtained. The protocol was registered in the PROSPERO International Prospective Register of Systematic Reviews (CRD42024576389). **Results**: The search produced nine relevant clinical trials with a total of 1345 COPD patients. Four of the trials incorporated obesity (BMI ≥ 30) as an inclusion criterion, while the other five had mixed samples, with patients presenting either overweight or obesity (four patients with BMI ≥ 25 and one with BMI ≥ 27). The risk of bias tool for randomised trials showed that all nine studies had a low risk of bias. Overall, these studies highlight the importance of overweight management and reject the use of extreme measures. Furthermore, they confirm the association between overweight/obesity and COPD, for which this condition is a risk factor, to a degree depending on the BMI. Four studies reported significant improvements in the clinical management of COPD patients following appropriate physical exercise. Specifically, one study observed that supervised exercise improved cardio-vascular performance; another, that observed that aquatic exercise increased maximal capacity, endurance and quality of life; another, that found cycling improved ventilatory performance; and the fourth, that observed exercise complementary to standard therapy in hospitalised obese COPD patients improved strength, exercise capacity and other perceived variables such as anxiety, mobility and dyspnoea. **Conclusions**: The therapeutic management of overweight COPD patients should include weight control, physical exercise and appropriate pharmacological treatment. Physical exercise is associated with improvements in endurance, exercise capacity, cardio-vascular performance, ventilatory performance and strength. In addition, the participants in these studies self-perceived clinical improvement. These findings justify the performance of further RCTs examining the role of physical exercise in patients with COPD and overweight/obesity, in order to improve their clinical outcomes and quality of life.

## 1. Introduction

Chronic obstructive pulmonary disease (COPD) is a respiratory condition that causes progressive airflow limitation. It is the third leading cause of mortality worldwide, accounting for 3.23 million deaths in 2019 [1]. According to the Global Initiative for Chronic Obstructive Pulmonary Disease 2023–2024, the condition results from a lifelong interaction between genotype and phenotype. Such interactions can damage the lungs and/or alter their normal development. The main environmental exposures associated with COPD are smoking and the inhalation of particulate matter and toxic gases from air pollution. In addition, idiosyncratic factors such as mutations in the SERPINA 1 gene, causing alpha-1-antitrypsin deficiency, may contribute to its appearance [2].

Overweight and obesity are characterised by an abnormal increase in body weight, usually due to excessive fat accumulation, a condition that is considered epidemic in the 21st century. In 2022, 2.5 billion adults aged 18 and older were classified as overweight (43% of the population, compared to 25% in 1990); of these individuals, more than 890 million were obese. In 1990, only 2% of young people (aged 5 to 19 years) were obese, but this figure has since climbed to 8% [3].

The impact of overweight has serious health implications for patients with COPD, particularly when central or abdominal obesity is involved [4]. Among the most notable effects are a reduction in forced vital capacity (FVC) [5] and an increase in respiratory load, meaning the respiratory system must work harder to meet the body’s oxygen demands [6].

Additionally, mild chronic systemic inflammation of adipose tissue can release pro-inflammatory cytokines that accelerate the progression of COPD and associated comorbidities [7]. Furthermore, the management of overweight may weaken the response to conventional treatments and limit physical mobility, thus reducing the patient’s ability to engage in pulmonary rehabilitation for COPD [8]. Lastly, and importantly, the number of comorbidities is higher in overweight COPD patients than in those without excess body weight [9]. The combined effect of these comorbidities and the complications arising in therapy can mean that these patients require palliative care [10].

When these clinical contexts coincide, a complex interaction occurs that can acutely worsen respiratory symptoms, complicate clinical management and reduce the patient’s quality of life [8]. Unfortunately, overweight is common among patients with COPD; according to recent data, the incidence of their joint presence ranges from 15% to 54% [11]. Other negative factors may include increased fatigue, exercise intolerance, and anxiety and depression, all of which hamper clinical management [12].

This difficult clinical context may be further complicated by the sudden worsening, or acute exacerbation, of COPD, which is quite common in patients suffering from overweight or obesity [6]. This exacerbation manifests as a worsening of respiratory symptoms, such as the onset of dyspnoea and increased coughing and sputum production, in a stable patient after 14 days of adjusted medication [2]. These outcomes heighten disease progression and comorbidities [13], severely impacting the quality of life and reducing patient survival [8].

The incidence of such complications appears to be rising [14]. It has been suggested that approximately 25% of patients with COPD currently present impaired lung function due to acute exacerbations [15], and obesity has been identified as one of the predictors of hospital readmission [16]. In this respect, clinicians have emphasised the importance of healthy lifestyle habits as a means of achieving controlled weight loss for COPD patients, who are at real risk of lung dysfunction due to malnutrition [17].

In view of these considerations, the present study was undertaken to examine how the degree of overweight or obesity can influence the therapeutic management of patients with COPD.

## 2. Materials and Methods

Design:

This systematic review with meta-analysis was conducted in accordance with the PRISMA (Preferred Reporting Items for Systematic Reviews and Meta-Analyses) guidelines.

Search strategy:

The Medline/PubMed database was searched using the following MESH descriptors: “(Pulmonary Disease, Chronic Obstructive OR COPD) AND (obesity OR overweight)”.

**Figure 1:**
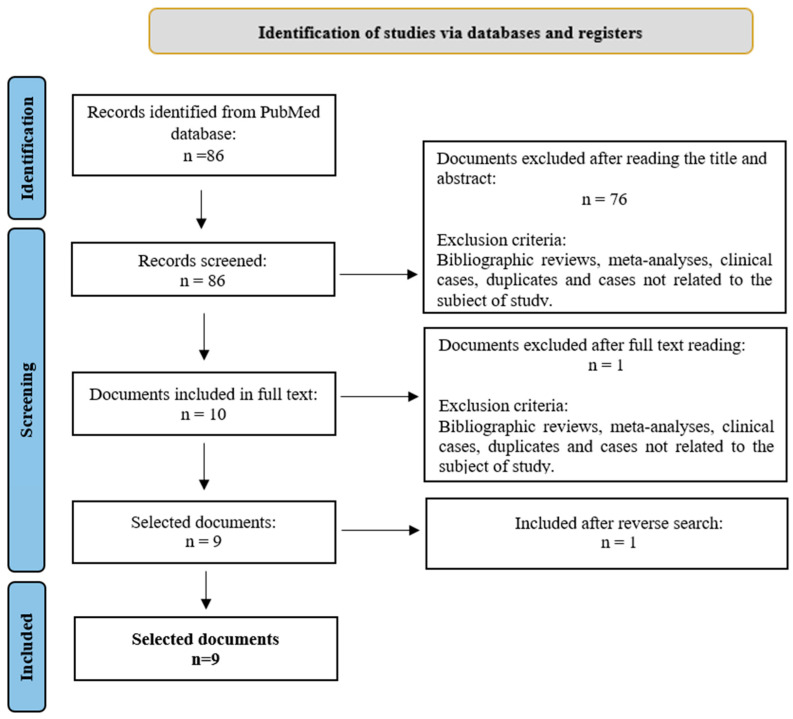
Identification and selection of clinical trials for the systematic review of the association between overweight/obesity and COPD.

The search was performed in October 2024. Two reviewers participated in the selection process (see Figure 1—Flowchart). The study was registered in the International Prospective Register of Systematic Reviews (PROSPERO) under the code CRD42024576389.

Eligibility criteria:

Inclusion criteria: clinical trials published in English with full-text access; no chronological restrictions; studies of COPD patients with a body mass index (BMI) ≥ 25 kg * m^−2^.

Exclusion criteria: doctoral theses; studies in which the population had other respiratory diseases.

Study selection process:

Two team members (S.C.-P. and A.M.C.-M.) independently conducted the search and study selection process. If any disagreements arose, a third investigator (G.A.C.-D.l.F.) was consulted. The article selection process consisted of four stages:

1. Review titles and abstracts;
2. Exclude articles failing to meet eligibility criteria;
3. Perform full-text reading and analysis;
4. Perform backward citation tracking on the selected articles.

Data extraction:

An ad hoc data collection form (Table 1) was developed to summarise the data extracted from each study, with the following information: lead author, publication year, study country, methodological design, sample details, study focus intervention, main outcomes and evidence level.

This information was then subjected to a descriptive analysis for the systematic review, followed by a random-effects meta-analysis to evaluate the effectiveness of an 8-week physical exercise programme in improving the results obtained by COPD patients in the 6-min walking test. Heterogeneity was assessed using the I^2^ statistic and the Cochrane RevMan Web tool.

Risk of bias assessment:

The risk of bias was independently evaluated by two reviewers (J.L.G.-U. and M.J.M.-J.) using the RoB2 and ROBINS-E tools [27,28] (see Table 2).

## 3. Results

### 3.1. Selection Process

The initial database search produced 752 articles, from which 214 duplicates were removed. After the title and abstract review, a further 372 articles were excluded for not meeting the inclusion criteria. From the remaining articles, 41 were considered potentially relevant and were evaluated in detail. Finally, 11 articles were selected for review (Figure 1).

### 3.2. Overweight, Obesity and COPD: A Complex Relationship

Au et al. [18] conducted a randomised clinical trial to assess the impact of a lifestyle intervention on the physical functional status of obese COPD patients. One group received usual care, while the other received an additional lifestyle intervention, including a self-directed video delivering the diabetes prevention programme. After the lifestyle intervention, the patients in this group were able to walk farther in the 6-min walking test, experienced less dyspnoea and achieved greater weight loss, compared to the control group. However, only the weight loss represented a clinically significant improvement.

Dupuis et al. [22] studied the impact of obesity on dyspnoea in COPD patients. The researchers compared different BMI categories and concluded that obesity was associated with severe obstructive sleep apnoea syndrome (OSAS), reduced expiratory reserve volume, increased forced expiratory volume in one second (FEV1), reduced carbon monoxide diffusion capacity (DLCO) and reduced rate constant for carbon monoxide uptake per alveolar volume (DLCO/VA). No statistically significant differences were found between BMI categories.

DeLapp et al. [21] compared short- and long-term outcomes for obese and non-obese COPD patients hospitalised due to exacerbation associated with influenza. In addition to standard treatment, the intervention group received oseltamivir medication. While the additional treatment did not produce a statistically significant difference in recovery, the obese patients had higher rates of comorbidities such as hypertension, hyperlipidaemia, diabetes mellitus, congestive heart failure and coronary artery disease. Nevertheless, mortality was also reduced in these patients.

In a clinical trial by Altinlas Dogan et al. [19], the study hypothesis was that treatment with the GLP-1RA liraglutide would improve lung function in obese COPD patients. The results showed that patients treated with this medication experienced significant weight loss, increased FVC and DLCO, and improved results in the COPD Assessment Test (CAT), which measures the impact of COPD on patients’ well-being and daily life.

### 3.3. Benefits of Physical Exercise in Patients with Overweight/Obesity and COPD

Ercin et al. [23] studied the effectiveness of supervised pulmonary rehabilitation programmes compared with a home exercise programme, for overweight or obese COPD patients. The first group performed an interval aerobic exercise programme in conjunction with the home exercise programme; the second group performed a continuous aerobic exercise programme in conjunction with the home exercise programme; and the third group performed only the home exercise programme. The researchers concluded that exercise capacity and health-related quality of life improved to a greater degree among the patients who performed either the continuous or the interval aerobic exercise programmes, in addition to the home exercise programme.

In the study by McNamara et al. [25], obese COPD patients took part in an 8-week water-based exercise training programme, and the results obtained were compared with those for patients who performed a similar land-based exercise programme. The water-based exercise group experienced greater improvements, in terms of reduced body weight, improved exercise capacity and enhanced health-related quality of life, than the patients who performed the land-based programme.

The study aim of McDonald et al. [24] was to determine the effect of weight reduction in obese COPD patients. The intervention involved a diet-induced energy restriction combined with resistance exercises. The researchers concluded that this programme generated a clinically significant improvement in body mass index (BMI), exercise tolerance and overall health status while maintaining muscle mass.

Ba et al. [20] studied the cardiopulmonary response of overweight COPD patients to an unloaded cycling exercise. The intervention consisted of exercising on an electrically braked cycle ergometer connected to software. The protocol included a resting period, followed by 2 min of unloaded cycling. Subsequently, the load was gradually increased until the patient’s oxygen consumption reached the levels corresponding to a healthy subject, or until the patient decided to stop. Forced vital capacity (FVC), forced expiratory volume in one second (FEV1) and total lung capacity (TLC) were measured with a body plethysmograph. The study found that hypoxaemia and overweight had differing impacts on heart rate and ventilatory responses to unloaded cycling. The results suggest that a better understanding of the relationship between increased ventilatory response and exercise may contribute to improving outcomes for overweight COPD patients enrolled in a rehabilitation programme.

Finally, Torres-Sánchez et al. [26] analysed the results obtained in a multimodal therapeutic programme, in addition to standard medical/pharmacological care, during the hospitalisation of obese patients with acute exacerbation of COPD. The sample consisted of a control group (standard care) and an intervention group (standard care plus the multimodal therapeutic programme). It was concluded that the intervention group experienced improvements in strength, exercise capacity, dyspnoea, quality of life and psychological well-being.

### 3.4. Meta-Analysis on the Effect of Exercise on Physical Capacity in COPD Patients

**Figure 2:**
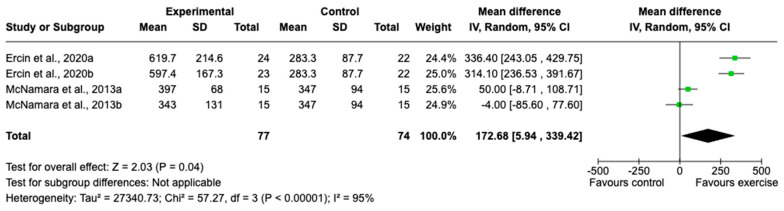
Forest plot of the effectiveness of exercise on the 6-min walking test.

A random effects meta-analysis of studies by McNamara et al. and Ercin et al. [23,25] was conducted to assess the impact of an 8-week exercise intervention on physical capacity, as measured by the 6-min walking test performed by an intervention group (n = 77) vs. a control group (n = 74). The exercise group had better outcomes, on average achieving a total distance of 339.42 metres, vs. 172.68 metres by the control group (95% CI: 5.94; *p* < 0.05). The Forest plot illustrating this result is shown in Figure 2.

## 4. Discussion

The relationship between overweight/obesity and COPD is complex and multifaceted, as both conditions interact in significant ways that impact patients’ health. The comorbidity of COPD and overweight ranges between 32 and 57%, and the prevalence of obesity in COPD patients is between 35 and 54% [29], making it a critical factor to consider. This association can be attributed to the fact that excess fat predisposes individuals to reduced lung distensibility, increased elastic load on inspiratory muscles and decreased exercise capacity [30]. However, studies suggest obesity may increase survival in COPD patients by up to 40% compared to those with low body weight [31], indicating the paradoxical nature of this relationship.

In the studies reviewed, lifestyle interventions aimed at improving physical condition and promoting weight loss resulted in enhanced quality of life. Symptoms like dyspnoea, fatigue and reduced exercise capacity, which are often associated with excess weight, are linked to a higher risk of mortality in COPD patients [32]. No dietary strategies or supplementation protocols have yet been shown to improve cardiorespiratory function directly. Therefore, much of the focus in current research revolves around body weight reduction, which has a clearer impact on lung function [33].

Although the body mass index (BMI) has been identified as a potential independent prognostic factor, the results of our analysis do not support this conclusion. The relationship between the BMI and mortality in COPD patients does not follow a linear pattern but instead forms a U-shaped curve, a pattern illustrating the obesity paradox, according to which, individuals with overweight or obesity may have lower mortality rates in certain conditions. In fact, obesity may even serve as a protective factor at specific BMI thresholds, underscoring the importance of considering the various phenotypes of COPD patients [34]. This paradox also explains the association with other comorbidities, such as FEV1 decline, reduced walking distance in the 6-min walk test, increased dyspnoea and higher rates of hospitalisation due to exacerbations [35,36,37]. The findings suggest that obesity may exacerbate these comorbidities or foster patterns of unhealthy behaviour [38,39]. Additionally, the coexistence of COPD and obstructive sleep apnoea syndrome (OSAS) can further impair exercise capacity, sleep quality and overall quality of life, significantly increasing the risk of mortality [40].

Given all these complexities, managing COPD patients with overweight/obesity is not a straightforward task. The results of our review indicate that there is a growing trend towards using pharmacological interventions to reduce weight and improve lung function. The complexity of these patients’ health status means it is essential to maintain their weight within safe limits, avoiding extremes of BMI both above and below the recommended range, thus minimising long-term risks [41]. One medication that may be relevant in both these respects is the GLP-1RA liraglutide, which several studies suggest may reduce the prevalence of exacerbation and also prevent complications related to excess weight [42,43].

Physical activity is of crucial importance in enabling these patients to address the challenges they face. Various types of exercise are known to be beneficial, but the studies included in this review particularly support aerobic exercise, whether on land or in water. This is unsurprising given that COPD has significant effects on lung function and in other areas [44], and aerobic exercise helps with weight loss, improves exercise capacity and enhances quality of life [45].

Nevertheless, other studies have observed that exercise alone might not be sufficient and recommend that dietary restrictions be incorporated. The combination of these two approaches can reduce the BMI, improve exercise tolerance and enhance overall health. Similarly, research has shown that exercise can partially counteract the extrapulmonary features of COPD [46]. Moreover, dietary adjustments also improve pulmonary function by reducing systemic inflammation [47], while dietary supplements may increase muscle mass [48]. This combination of approaches is particularly promising for obese COPD patients, as it enhances the overall effectiveness of treatment [49].

Some studies have suggested that the ventilatory functional response to lower extremity exercise is particularly important for the rehabilitation of COPD patients with overweight. This improvement is thought to result from the better efficiency of the respiratory muscles and from thoracic wall mobilisation, leading to enhanced oxygenation [50]. Additionally, this form of exercise generates positive adaptations, reducing fatigue, decreasing afferent feedback from limb muscles, reducing ventilatory demand and alleviating exertional dyspnoea in COPD patients [51,52].

Finally, pulmonary rehabilitation, especially in patients with exercise intolerance, offers significant benefits, including enhanced cardiorespiratory function, increased muscular strength and endurance, and reduced symptoms of dyspnoea [53]. Implementing pulmonary rehabilitation during COPD exacerbations, particularly in the hospital setting, not only facilitates continued rehabilitation after discharge but also reduces the risk of hospital readmissions and decreases mortality [54,55].

### Limitations and Future Research

Despite the promising results obtained in this systematic review and meta-analysis, several limitations must be considered. The heterogeneity of study designs and interventions represented in the studies considered makes it challenging to draw definitive conclusions about the optimal type, duration and long-term intensity of exercise interventions. Additionally, while the meta-analysis demonstrated significant improvements in physical capacity, the long-term effects of exercise and weight management on COPD outcomes remain unclear. Therefore, an in-depth analysis of combination therapies would strengthen the clinical implications.

Another important limitation is that the number of studies is low and, therefore, there are topics of interest that are not addressed in them. Variables such as the obesity paradox or the fat-free mass index may be of interest as they may reflect interesting aspects of the exercise capacity and nutritional status of COPD patients. Due to the low number of studies included in our meta-analysis, the results obtained should be taken with caution.

In short, future research should be conducted with a focus on larger, more diverse patient populations and long-term follow-up studies, in order to better understand the lasting impact of these interventions. Furthermore, more intervention studies with similar interventions and outcomes should be performed in different countries to increase future meta-analysis information. Finally, exploring the combined effect of pharmacological treatments and lifestyle interventions on both obesity and COPD is an area of ongoing research that holds significant potential for improving patient outcomes.

## 5. Conclusions

The interaction between COPD and overweight in patients creates a significant challenge for healthcare professionals, as their association is complex and paradoxical. It seems that overweight is associated with the presence of comorbidities; however, patients with excess weight may have a better ventilatory response and a lower degree of inflation and air trapping.

Therefore, an integrated approach should include weight control, physical exercise aimed at pulmonary rehabilitation, and appropriate pharmacological treatment, with the ultimate aim of improving patients’ clinical outcomes and quality of life.

## Figures and Tables

**Table 1:** Characteristics of the studies analysed.

Author, _Year	Type of Study	Sample	Intervention	Results
Au et al., 2023 [18].	Randomised clinical trial.	346 in the intervention group and 338 in the control group.	The intervention was a self-directed video programme for lifestyle balance aimed at promoting moderate weight loss, moderate-intensity physical activity, and behavioural self-management techniques. An informational session was held with a health advisor, followed by 12 weekly video sessions. Participants could track their weight, diet and physical activity on MyFitnessPal or in tracking booklets provided as part of the study, and received a step tracker. Health advisors sent standardised reminders through MyFitnessPal messages or email every 2 weeks for 52 weeks.	The participants with COPD and overweight who were assigned to the intervention group walked more (adjusted difference, 42.3 feet (95% CI: 7.9–76.7 feet; *p* = 0.02), had less dyspnoea at the end of the 6-min walk test (adjusted difference, −0.36 [95% CI, −0.63 to −0.09]; *p* = 0.008), and had lost more weight (adjusted difference, −1.34 kg [95% CI, −2.33 to −0.34 kg]; *p* = 0.008) compared to the control group. There were no statistically significant differences in walking distance (98.4 feet) or dyspnoea (1 unit), but there were differences in weight loss (3% and 5%).
Altintas Dogan et al., 2022 [19].	Randomised clinical trial.	40 patients with obesity and COPD, randomly assigned to receive medication.	The effect of liraglutide on lung function in patients with obesity and COPD was investigated. In the intervention, liraglutide (3 mg subcutaneous) was administered to the intervention group and a placebo (subcutaneous) to the control group in a 1:1 ratio via an electronic device for 40 weeks. The liraglutide dose was adjusted weekly in increments of 0.6 mg/day until reaching 3 mg/day or the corresponding placebo by week 4, and this dose was maintained until week 40. At 40 weeks, participants were evaluated to determine the full effect of liraglutide (3 mg) and weight loss. Four weeks later (week 44), participants were reassessed to determine the effect of weight loss without continuous exposure to liraglutide.	The use of liraglutide resulted in significant weight loss, an increase in FVC and carbon monoxide diffusion capacity, and an improvement in the CAT score. No significant changes were observed in FEV1, FEV1/FVC or the 6-min walk test. After 40 weeks of treatment with liraglutide, improvements in some measures of lung function were recorded.
Ba et al., 2015 [20].	Clinical trial.	63 male subjects of the same age (18 sedentary subjects in good health; 14 hypoxaemic COPD patients with normal weight; and 31 overweight patients, including 12 who were hypoxaemic).	The intervention consisted of exercise on a cycle ergometer connected to software. The protocol included a 5-min rest period followed by a 2-min pedalling period without load. Then, the ramp was increased until the oxygen consumption reached the level expected in healthy subjects or when the patient decided to stop. In all subjects, FVC, FEV1, and TLC were measured using a pressure displacement plethysmograph. Ventilation was measured using a volumetric rotor transducer.	In the hypoxaemic patients, there was a greater increase in heart rate at the plateau. In the overweight patients, there was an increase in minute ventilation and respiratory rate responses. However, the increase in heart rate at the plateau during pedalling without load was not correlated with changes in heart rate at the ventilatory threshold or with maximum oxygen consumption. Similarly, pedalling without load before exercise was not associated with an increased heart rate during incremental exercise.
DeLapp et al., 2020 [21].	Randomised clinical trial.	301 patients.	Analysis of patients hospitalised for acute exacerbation of COPD. Oral treatment with oseltamivir at hospital admission was compared with routine clinical care for adults hospitalised with symptoms consistent with acute LRTI during three consecutive influenza seasons (2010–2011, 2011–2012, and 2012–2013). The outcomes evaluated included time to clinical stability, length of hospital stay and mortality.	The mortality of COPD patients with obesity was <1% at discharge, 3% at 30 days, 7% at six months, and 8% at one year. The mortality of patients without obesity was <1% at discharge, 3% at 30 days, 18% at six months, and 28% at one year. Patients with obesity had a median time to clinical stability of two days, compared to three days for those without obesity. Compared to patients without obesity, the one-year mortality odds ratio was significantly reduced (adjusted odds ratio [aOR]: 0.18; 95% CI: 0.06–0.58; *p* = 0.004). The corresponding values for six-months mortality were aOR: 0.28; 95% CI: 0.09–0.89; *p* = 0.031. In the sensitivity analysis, excluding patients with BMI < 21, the one-year mortality remained significant (aOR: 0.24; 95% CI: 0.07–0.87; *p* = 0.030; n = 255).
Dupuis et al., 2024 [22].	Clinical trial.	127 COPD patients with a BMI ≥ 18.5 kg/m^2^.	Dyspnoea in COPD patients was assessed in relation to their BMI, related factors and obesity. A statistical analysis of dyspnoea was conducted in subgroups according to BMI and GOLD spirometric stages. Dyspnoea was evaluated using the mMRC scale. Pulmonary function was tested and emphysema was quantified using computed tomography. Laboratory parameters such as haemoglobin, eosinophils, fibrinogen, total protein and the N-terminal prohormone of brain natriuretic peptide were determined.	No significant differences were observed between the BMI categories regarding dyspnoea. Dyspnoea intensity was associated with more severe pulmonary insufficiency. No association was found between the mMRC scale and the exacerbation rate at the 3rd and 12th months prior to inclusion. The mMRC score correlated with a lower forced expiratory volume in the first second (FEV1) (correlation coefficient: −0.427; *p* < 0.0001), DLCO, and DLCO/VA (correlation coefficients of −0.427; *p* < 0.0001 and −0.317; *p* = 0.002, respectively). The mMRC score was also correlated with a higher emphysema score (correlation coefficient: +0.343; *p* = 0.0002).
Ercin et al., 2020 [23].	Randomised clinical trial.	72 COPD patients with overweight or obesity.	The three groups performed a home exercise programme. Additionally, Group 1 performed an interval exercise programme, and Group 2 performed a continuous exercise programme. Anthropometric measurements were obtained, cardiopulmonary function was tested by the 6-min walk test, dyspnoea scores and the modified Borg fatigue index were calculated, and the SGRQ and HADS questionnaires were completed.	A continuous or interval aerobic exercise programme performed in conjunction with a home exercise programme improved exercise capacity and health-related quality of life, and reduced levels of anxiety and depression in overweight and obese COPD patients. The groups that performed continuous or interval aerobic exercise recorded greater improvements in cardiopulmonary exercise testing, walking distance, mental health and quality of life compared to the home exercise group (*p* < 0.001). The interval group achieved a significant reduction in modified Borg dyspnoea and leg fatigue during cardiopulmonary exercise testing compared to the continuous aerobic exercise and home exercise groups (*p* < 0.001). Additionally, Borg dyspnoea and leg fatigue during training were lower in the interval training group than in the continuous training group (*p* < 0.05).
McDonald et al., 2016 [24].	Clinical trial.	28 patients with obesity and COPD.	Participants were prescribed a low-energy diet ranging from 3850 to 5000 kJ/day, or up to 5900 kJ/day for those with a BMI > 40 kg/m^2^. Participants then performed a home-based strength training programme for the upper and lower limbs, 3 days a week, with a rest day between training sessions.	At the start of the study, the mean BMI of the participants was 36.3 kg/m^2^. By the end, this had decreased by 2.4 kg/m^2^ (1.1 kg/m^2^; *p* < 0.0001). Skeletal muscle mass remained unchanged. Health status improved significantly, with a mean change in the total SGRQ score of 9.6 ± 12.7 units (*p* = 0.0005). The participants achieved a significant reduction in the BODE index, of 1.4 ± 1.2 units (*p* < 0.0001) by the end of the 3-month intervention. There were no changes in systemic inflammatory biomarkers. Despite the improvement in FVC, there were no changes in any other pulmonary function parameters.
McNamara et al., 2013 [25].	Randomised clinical trial.	53 COPD patients with obesity and other physical comorbidities.	The study compared the effects of aquatic and land-based exercise, vs. a control group that did not exercise, over a period of eight weeks. Participants were randomly assigned to one of three groups: water exercise training, land exercise training or control (no exercise). Randomisation was stratified according to the limiting factor in the 6-min walk test (i.e., dyspnoea or physical comorbidity) and BMI (≥32 kg/m^2^). Due to the nature of the exercise interventions, it was not possible to blind the therapist or participants to their allocation.	Obesity in COPD was associated with increased dyspnoea, worse health-related quality of life, higher levels of fatigue and limitations in physical performance. Only the water exercise group achieved a significant improvement in exercise capacity, with a mean ± SD change in 6MWD of 41 ± 36 m (*p* = 0.01), in ISWT of 60 ± 48 m (*p* = 0.01), and in ESWT of 476 ± 400 m (*p* = 0.01).
Torres-Sánchez et al., 2015 [26].	Randomised clinical trial.	49 patients were randomised to the intervention group or the control group.	The results obtained from a pulmonary rehabilitation programme performed in conjunction with usual care for COPD patients hospitalised for exacerbation were compared with those of a control group receiving only usual care. The programme included deep breathing exercises and limb exercises. Anthropometric measurements and medical history data were recorded. Spirometry was performed on all subjects, and pulmonary, physical and perceived measures were evaluated at hospital admission and discharge.	Pulmonary variables improved significantly in both groups (*p* < 0.05). Physical variables improved significantly in the intervention group. Lower limb strength worsened significantly in the control group (*p* < 0.05). Dyspnoea scores improved significantly in both groups (2.20 ± 2.6; *p* < 0.001 in the intervention group vs. 3.6 ± 2.21; *p* < 0.001 in the control group), with no significant differences between their outcomes (*p* = 0.785).

6MWD: 6-min walk test; BMI: Body mass index; BODE: Body max index, airflow Obstruction, Dyspnoea and Exercise; CAT: COPD Assessment Test; COPD: Chronic obstructive pulmonary disease; DLCO: Diffusing capacity for carbon monoxide; DLCO/VA: Rate constant for carbon monoxide uptake; ESWT: Endurance shuttle walk test; FEV1: Forced expiratory volume in 1 s; FVC: Forced vital capacity; GOLD: Global Initiative for Chronic Obstructive Lung Disease; HADS: Hospital Anxiety and Depression Scale; ISWT: Incremental shuttle walk test; LRTI: Lower respiratory tract infections; mMRC: Modified medical research council; SD: Standard deviation; SGRQ: Saint George respiratory questionnaire; TLC: Total lung capacity.

**Table 2:** Critical appraisal results based on the Cochrane risk of bias tools for randomised trials (RoB2) and for non-randomised follow-up studies of exposure effects (ROBINS-E).

**Study**	**Domain 1**	**Domain 2**	**Domain 3**	**Domain 4**	**Domain 5**
**Randomised clinical trials**					
Altintas Dogan et al., 2022 [19].	1.1 Yes	2.1 PY	3.1 Yes	4.1 No	5.1 Yes
	1.2 Yes	2.2 PY		4.2 No	
	1.3 No	2.6		4.3 Yes	
	Low	Some concerns	Low	Low	Low
Au et al., 2023 [18].	1.1 Yes	2.1 PY	3.1 Yes	4.1 No	5.1 Yes
	1.2 Yes	2.2 No		4.2 No	
	1.3 No	2.6 Yes		4.3 Yes	
	Low	Low	Low	Low	Low
Ercin et al., 2020 [23].	1.1 Yes	2.1 PY	3.1 Yes	4.1 No	5.1 Yes
	1.2 Yes	2.2 PY		4.2 No	
	1.3 No	2.6 Yes		4.3 No	
	Low	Some concerns	Low	Low	Low
McNamara et al., 2013 [25].	1.1 Yes	2.1 PY	3.1 Yes	4.1 No	5.1 Yes
	1.2 Yes	2.2 No		4.2 No	
	1.3 No	2.6 Yes		4.3 Yes	
	Low	Low	Low	Low	Low
Torres-Sánchez et al., 2016 [26].	1.1 Yes	2.1 PY	3.1 Yes	4.1 No	5.1 Prob. Yes
	1.2 Yes	2.2 PY		4.2 No	
	1.3 No	2.6 Yes		4.3 No	
	Low	Some concerns	Low	Low	Low
**Non Randomised**					
**Study**	**Domain 1**	**Domain 2**	**Domain 3**	**Domain 4**	**Domain 5**
Ba et al., 2015 [20].	1.1 Yes	2.1 Yes	3.1 Yes	4.1 Prob. No	5.1 Prob. Yes
	1.2 Yes	2.2 No			5.2 Yes
	1.3 No				5.3 Prob. Yes
	1.4 No				
	Low	Low	Low	Low	Low
DeLapp et al., 2020 [21].	1.1 Yes	2.1 Yes	3.1 Yes	4.1 Prob. No	5.1 Yes
	1.2 Yes	2.2 No			5.2 Yes
	1.3 No				5.3 Yes
	1.4 P. No				
	Low	Low	Low	Low	Low
Dupuis et al., 2024 [22].	1.1 Yes	2.1 Yes	3.1 Yes	4.1 No	5.1 Prob. Yes
	1.2 Yes	2.2 No			5.2 Yes
	1.3 No				5.3 Prob. Yes
	1.4 Prob. No				
	Low	Low	Low	Low	Low
McDonald et al., 2016 [24].	1.1 Yes	2.1 Yes	3.1 Yes	4.1 No	5.1 Yes
	1.2 Yes	2.2 No			5.2 Yes
	1.3 No				5.3 Yes
	1.4 No				
	Low	Low	Low	Low	Low

Prob: Probably.

## Data Availability

Not applicable.
